# Nutrition and its role in epigenetic inheritance of obesity and diabetes across generations

**DOI:** 10.1007/s00335-020-09839-z

**Published:** 2020-04-30

**Authors:** Daniela Kaspar, Sieglinde Hastreiter, Martin Irmler, Martin Hrabé de Angelis, Johannes Beckers

**Affiliations:** 1 Institute of Experimental Genetics, Helmholtz Zentrum München GmbH, Neuherberg, Germany; 2 Chair of Experimental Genetics, Technische Universität München, Weihenstephan, Germany; 3 Deutsches Zentrum für Diabetesforschung E.V. (DZD), Neuherberg, Germany

## Abstract

Nutritional constraints including not only caloric restriction or protein deficiency, but also energy-dense diets affect metabolic health and frequently lead to obesity and insulin resistance, as well as glucose intolerance and type 2 diabetes. The effects of these environmental factors are often mediated via epigenetic modifiers that target the expression of metabolic genes. More recently, it was discovered that such parentally acquired metabolic changes can alter the metabolic health of the filial and grand-filial generations. In mammals, this epigenetic inheritance can either follow an intergenerational or transgenerational mode of inheritance. In the case of intergenerational inheritance, epimutations established in gametes persist through the first round of epigenetic reprogramming occurring during preimplantation development. For transgenerational inheritance, epimutations persist additionally throughout the reprogramming that occurs during germ cell development later in embryogenesis. Differentially expressed transcripts, genomic cytosine methylations, and several chemical modifications of histones are prime candidates for tangible marks which may serve as epimutations in inter- and transgenerational inheritance and which are currently being investigated experimentally. We review, here, the current literature in support of epigenetic inheritance of metabolic traits caused by nutritional constraints and potential mechanisms in man and in rodent model systems.

## Introduction

Evidence that phenotypes acquired during life affect the offspring’s health across generations has increased over the past years. In particular, it has been suggested that parental over- as well as undernutrition impact on the metabolic health of the offspring. In this article we aim to review the existing literature on the epigenetic inheritance of phenotypes associated with parental nutrition on metabolic health in subsequent generations. With the term epigenetic inheritance, we refer to any mode of inheritance that is transmitted via gametes but that is not encoded in the nucleotide sequence of the DNA. This type of epigenetic information is also referred to as *epimutation* (Jablonka 1995). Methylation of DNA, chemical modifications of histones, and different species of RNAs are well-studied molecular marks of epimutations. However, clear functional data demonstrating causal relations between epimutations in gametes and associated epigenetic inheritance of traits across generations in mammals are still sparse (Irmler et al. 2020).

Within the literature there is occasionally some fuzziness in the use of the terms inter- and transgenerational epigenetic inheritance (Deans and Maggert 2015). Therefore, we want to define as clearly as possible what we and many others mean when we use these terms. *Intergenerational epigenetic inheritance* refers to a parentally (F0) acquired phenotype (e.g., from exposure to external factors such as diets or active compounds) that causes a distinct phenotypic alteration also in the next generation (F1) (Daxinger and Whitelaw 2012). In order to classify as intergenerational epigenetic inheritance, the phenotype must be transmitted via an epimutation in the maternal or paternal gametes. In this definition, intergenerational epigenetic inheritance is clearly distinct from other modes of non-genetic inheritance such as social interactions with parents, learned behaviors or shared microbiomes. Also, the exposure of embryos in utero to maternal effects and of newborns to maternal milk, and its effects on the individual do not represent a form of intergenerational epigenetic inheritance. Instead, from the viewpoint of the (epi-)geneticist it is the generation of exposed embryos or newborns that should be regarded as the F0 generation. However, in most publications which examine in utero exposure, authors refer to the maternal generation that receives a certain diet as F0 and the embryos they carry as F1. In the latter case, phenotypic alterations in F1 may also result from the exposure in utero. When we use the term intergenerational epigenetic inheritance, we always refer to epimutations associated with parental gametes (sperm or oocyte) that persist through or are somewhat resistant to the first cycle of epigenetic reprogramming that occurs in the mammalian preimplantation embryo (Schaefer and Nadeau 2015). When we use the term *transgenerational epigenetic inheritance* we refer to epimutations that, in addition, also persist through the second round of reprogramming that occurs in the primordial germ cells later in embryogenesis (Hajkova 2002). Consequently, transgenerational epigenetic inheritance is associated with the inheritance of acquired traits from the parental F0 generation (that was exposed to environmental challenges) to at least the generation of grand-children (F2) and possibly also subsequent generations. Although the inheritance of an acquired phenotype from F0 to F2 is a necessary condition of transgenerational inheritance, it is, however, not a sufficient criterion. For example, an intergenerationally inherited epigenetic phenotype may again affect epimutations in the gametes of the F1 generation. Thus, F2 animals may present an epigenetic phenotype, but they do so as a result of exposure in the F1 generation and not as a result of transgenerational epigenetic inheritance. We take the freedom to use the term *epigenetic inheritance across generations* when we either mean both modes of epigenetic inheritance or when we discuss experiments in which the distinction between inter- and transgenerational epigenetics was unclear or not even considered.

Although there are currently about 400 genetic loci that have been associated with obesity and diabetes, they explain only a minor fraction of the heritability of these metabolic disorders (Fuchsberger et al. 2016). Instead, in the contemporary environment, which is rich in energy-dense diets, epigenetic inheritance may be one of the important factors that contribute to this pandemic of obesity and diabetes, which we are experiencing since the 1960s. It is unlikely that genetic drift over two to three generations in man could be responsible for the pandemic. Instead, it has been suggested that our thrifty epigenotype (which is an ancient trait) primes us and our progeny to become increasingly obese when energy-rich diets are abundant (Stoger 2008).

Below we summarize the current literature on epidemiological studies and mammalian models that examine epigenetic inheritance of metabolic traits following dietary constraints. For this, we attempt to be as comprehensive as possible and cover most relevant studies on inter- and transgenerational inheritance published, so far. Since there are a plethora of studies dealing with effects of in utero exposure to diets, we needed to be more selective for this topic. In Table 1 we provide a comprehensive overview of studies that examine diet-related inter- and transgenerational inheritance in rodent models. Finally, we give an overview of the literature that studies the molecular basis of epimutations associated with nutrition. With the term diet we refer to nutrition in general, including also some specific food supplements that may interfere with epigenetic mechanisms.

**Table 1:** Studies in rodents examining diet-related effects and their epigenetic inheritance

Inheritance	Species	Nutritional exposure	Exposed parent	Analyzed generation	Analyzed sex	Inheritance lineage			IVF	Epigenetic mark	References
						F0 —> F1	F1 —> F2	F2 —> F3			
In utero	Rat	LPD	M	F1	m + f	M			N	–	Sayer et al. (2001)
	Rat	LPD	M	F0–F1	m	M			N	–	Passos et al. (2000)
	Rat	CR	M	F0–F1	m + f	M			N	–	Desai et al. (2007)
	Mouse	LPD	M	F1	m	M			N	–	Crossland et al. (2017)
	Mouse	LPD	M	F1	m	M			N	meDNA	Balasa et al. (2011)
	Rat	LPD	M	F1	m	M			N	–	Cavariani et al. (2019)
	Rat	LPD	M	F1	m	M			N	–	Rodriguez-Gonzalez et al. (2014)
	Rat	LPD	M	F1	m	M			N	–	Toledo et al. (2011)
	Mouse	CR	M	F1	m + f	M			N	–	Jimenez-Chillaron et al. (2005)
	Mouse	HFD	M	F1	m + f	M			N	meHistone, acHistone	Masuyama and Hiramatsu (2012)
	Rat	HFD	M + P	F1	m	P			N	–	Buckley et al. (2005)
	Mouse	HFD	M	F1	m	M			N	meDNA	Cannon et al. (2014)
	Mouse	HFD	M	F0–F1	m + f	M			N	meDNA	Ge et al. (2014)
	Mouse	Methyl supplementation	M	F1	m + f	M			N	–	Wolff et al. (1998)
	Mouse	CR	M	F1	m	M			N	meDNA, acHistone	Zelko et al. (2019)
	Rat	CR	M	F1	m	M			N	acHistone	Zhu et al. (2016)
Intergenerational	Mouse	LPD	P	F1	m + f	P			N	meDNA	Carone et al. (2010)
	Mouse	CR	M	F1–F2	m	M	P		N	meDNA	Radford et al. (2014)
	Mouse	CR	M	F1–F2	m + f	M	M, P, M + P		N	–	Jimenez-Chillaron et al. (2009)
	Mouse	CR	M	F1–F2	m + f	M	M, P, M + P		N	–	Kaczmarek et al. (2016)
	Rat	CR	M	F1–F2	m + f	M	M, P		N	–	Harper et al. (2014)
	Mouse	LPD	P	F0–F1	m	P			Y	smallRNA	Sharma et al. (2016)
	Mouse	HFD	M	F1–F2	f	M	M		N	meHistone, acHistone	Masuyama et al. (2015)
	Mouse	HFD	M	F0–F2	m	M	M		N	–	Graus-Nunes et al. (2015)
	Rat	HFD	M	F0–F2	m + f	M	M		N	–	Huang et al. (2017)
	Rat	HFD	P	F0–F1	f	P			N	meDNA	Ng et al. (2010)
	Rat	HFD	P	F1	f	P			N	–	Ng et al. (2014)
	Mouse	HFD	P	F0–F1	m	P			N	–	Fullston et al. (2015)
	Mouse	HFD	P	F1	m + f	P			N	meDNA	Wei et al. (2014)
	Mouse	HFD	M + P	F0–F1	m + f	M, P, M + P			Y	–	Huypens et al. (2016)
	Mouse	HFD	M	F0–F2	m + f	M	M, P, M + P		N	meDNA	Dunn and Bale (2009)
	Mouse	± folic acid	M	F0–F2	m	M	P		N	meDNA	Ly et al. (2017)
	Rat	Methyl depletion	P	F1	m	P			N	–	McCoy et al. (2018)
	Mouse	Methyl supplementation	M	F0–F2	m + f	M	P		N	–	Cropley et al. (2006)
	Mouse	HFD	M	F1–F2	m + f	M	P		Y	tsRNAs	Sarker et al. (2019)
	Mouse	HFD	P	F0–F1	m	P			N	lncRNAs	An et al. (2017)
Transgenerational	Mouse	LPD	M	F1–F3	m	M	M	M	N	–	Frantz et al. (2011)
	Rat	CR	M	F1–F3	f	M	M	M	N	–	Nowacka-Woszuk et al. (2018)
	Rat	LPD + CR	M + P	> 50 generations	m + f	F0 —> F50 M + P			N	meHistone, acHistone	Hardikar et al. (2015)
	Mouse	HFD	M	F3	m + f	M	P	P	N	–	Dunn and Bale (2011)
	Mouse	HFD	P	F0–F3	m + f	M	P	P	Y	meDNA	Sarker et al. (2018)
	Mouse	HFD	P	F0–F2	m + f	P	M, P		N	meDNA	Fullston et al. (2013)
	Mouse	HFD	P	F0–F2	m + f	P	M, P		N	–	Fullston et al. (2012)
	Rat	HFD	P	F0–F2	m + f	P	P		N	meDNA, sncRNAs	de Castro et al.(2016)
	Rat	HFD	P	F2	f	P	P		N	–	de Castro et al. (2019)
	Mouse	HFD/HSD	M	F0–F3	m + f	M	P	P	Y	–	Ferey et al. (2019)
	Rat	Vinclozolin	M	F0–F3	m	M	P	P	N	acHistone, meDNA, sncRNA	Ben Maamar et al. (2018)
	Rat	BPA	M	F1–F3	m + f	M	M + P	M + P	N	meDNA	Manikkam et al. (2013)
	Rat	BPA	M	F1–F3	m	M	P	P	N	–	Salian et al. (2009)

Comprehensive overview of published studies that examine inter- and transgenerational inheritance of diet-related effects in rodent models and selection of studies of in utero effects

*HFD* high-fat diet, *LPD* isocaloric low protein diet, *CR* caloric restriction, *BPA* bisphenol A, *HFD/HSD* high-fat and high sugar diet, *M* maternal, *P* paternal, *M + P* parental, *m* males, *f* females, *m + f* males and females, *Y* yes, *N* no, *meDNA* DNA methylation, *meHistone* Histone methylation, *acHistone* Histone acetylation, *sncRNA* small non-coding RNA, *tsRNA* tRNA derived small RNA, *lncRNA* long non-coding RNA

### Epidemiological studies in humans support the concept of intergenerational inheritance

Epidemiological studies in humans provide the opportunity to monitor effects of nutrition on health parameters also across generations. The impact of human obesity and type 2 diabetes as well as of factors such as physical activity on epigenetic mechanisms that control gene expression across generations was recently reviewed (Ling and Ronn 2019). Below we examine in more detail retrospective data from calamities such as famines as well as prospective data from human cohorts. We focus on the role of parental malnutrition for the predisposition to develop the metabolic syndrome in the following generations.

#### Retrospective studies: famines and historical records

Numerous studies evaluated data from the Dutch famine birth cohort of the hunger winter 1944/45. Overall, they revealed an association between intrauterine exposure to maternal undernutrition and the development of the metabolic syndrome including obesity, elevated blood glucose levels, and decreased glucose tolerance in later life of people born during the famine (de Rooij et al. 2006; Lumey et al. 2009; Ravelli et al. 1998). Furthermore, persistent changes in DNA cytosine methylation of several genes were found in blood samples from adult males that were undernourished in utero. Many of these genes were associated with pathways related to growth and metabolism (Heijmans et al. 2008; Tobi et al. 2009, 2014). These data agree with an impact of intrauterine and early postnatal undernutrition on metabolic health in adulthood. Interestingly, the prenatal undernutrition not only affected the generation born during the hunger winter, but also their offspring. This latter generation (progeny of parents that were born during the Dutch hunger winter) also displayed higher rates of obesity and increased neonatal adiposity, compared to offspring of prenatally well-nourished parents. The observations gained from detailed analyses of the Dutch famine birth cohort agree with the hypothesis that the metabolic health of parents potentially affects their offspring’s health (Painter et al. 2008; Veenendaal et al. 2013). However, sperm and oocytes of the parental generation were not archived at that time, precluding the study of epigenetic modifications in gametes as potential causative epimutations.

Effects of prenatal undernutrition on metabolic health later in adulthood were also studied in people that were born during the Chinese famine that occurred from 1959 to 1961. These studies reported overweight, type 2 diabetes, hyperglycemia, metabolic syndrome, and schizophrenia more frequently among adults born during the years of the famine compared to people born in the years after the famine. These data support direct effects of the in utero undernutrition. However, uncontrolled age differences of the analyzed probands of famine and post-famine birth groups during the evaluation period may also play a role (Li and Lumey 2017). An indication for epigenetic inheritance from one generation to the next came from a study examining the metabolic health in the generation that followed the parents born during the Chinese famine. Also they had an increased risk of hyperglycemia, similar to the second generation following the Dutch hunger winter. Although these observations may suggest the intergenerational transmission of a parentally acquired metabolic phenotype, potential causative epimutations were not examined (Li et al. 2017).

Another well-known study utilized historical records on food availability to associate mortality rates in residents of the Swedish town Överkalix as well as in their children and grand-children. One of the intriguing findings was that overnutrition during the slow growth period of childhood (girls 8 to 10 years, boys 9 to 12 years) had a profound effect on cardiovascular and diabetes-related deaths on filial and grand-filial generations through the male lineage (Kaati et al. 2002). In this cohort, evidence for transgenerational epigenetic inheritance in humans due to dietary alterations was described.

In the past decades Pima Indians and the citizens of Nauru represented populations with the highest increase of diabetes prevalence rates worldwide. It has been postulated that genetic predisposition together with sudden environmental changes such as processed food, calorie-dense diets (so-called cocacolarization) and a sedentary lifestyle were responsible for this dramatic progress (Bennett 1999; Zimmet et al. 1982). But so far, only very few studies address the impact epimutations could have on the rapidly increasing diabetes prevalence (Rosario et al. 2014). In particular, there is unfortunately no detailed analysis of inter- or transgenerational epigenetic inheritance in these populations available.

#### Prospective studies of human cohorts

In contrast to retrospective studies outlined above, prospective studies in man have the advantage that they are specifically designed to address certain research questions and they have lent additional support to the idea that parental exposure to diverse environmental factors can affect the offspring’s metabolic health.

For example, the Avon Longitudinal Study of Parents and Children (ALSPEC) showed that BMI was higher in 9-year-old sons the earlier the father started smoking and despite no significant association with BMI, daughters showed a reduction in total lean mass (Northstone et al. 2014; Pembrey et al. 2006). Grand-maternal smoking during pregnancy on the other hand, resulted in increased birthweight, body length at birth, and BMI in grandsons, but not granddaughters (Miller et al. 2014). A study in Norwegian men found that risk of obesity increased with higher paternal, but not maternal age at birth (Eriksen et al. 2013). Moreover, as shown in the Newborn Epigenetics STudy (NEST) cohort, paternal obesity altered small RNA content and DNA methylation in human spermatozoa (Donkin et al. 2016) as well as DNA methylation in umbilical cord blood leukocytes in offspring of these obese individuals (Soubry et al. 2015).

Compared to the historical famines and their adverse influence on metabolic health, moderate fasting and caloric restriction were repeatedly reported to have beneficial effects on longevity and metabolic health (Di Francesco et al. 2018). Six months of caloric restriction significantly improved health through reduced body weight and metabolic rate (Heilbronn et al. 2006) and also time-restricted feeding in which probands consumed their entire daily food allowance within a period of 4–12 h, showed improved health parameters (Gabel et al. 2018; Gill and Panda 2015; Tinsley et al. 2017). This was especially the case if feeding was carried out within the first half of the day and even when probands were suffering from metabolic syndrome (Wilkinson et al. 2020). Nevertheless, inheritance of these improved health markers has not been investigated yet.

Not so surprisingly, parental physical exercise can have a significant impact on the offspring as well (Denham 2018). Recent findings indicate that certain circulating miRNAs in blood are controlled by exercise (Bye et al. 2013; Nielsen et al. 2014; Wardle et al. 2015) and changes in genome-wide DNA methylation are detectable also in human spermatozoa (Denham et al. 2015). Such epimutations may provide tangible marks and molecular mechanisms of epigenetic inheritance across generations (see “Epimutations triggered by diets” section). Also, maternal exercise during pregnancy was linked to a reduced obesity risk in offspring (Mourtakos et al. 2015) and differentially methylated regions in imprinted genes in the offspring (McCullough et al. 2015). However, these observations could also be attributed to the in utero exposure of the embryo and not to epigenetic inheritance via gametes.

Even in the prospective human epidemiological studies it is an inherent difficulty to discern associations from causalities. An important complication of epidemiological studies, in general, is that they are often accompanied by additional confounding factors. For example, famines like those mentioned above, may also involve traumatic events, which were shown to also affect the offspring’s health (Yehuda et al. 2014; Mehta et al. 2019). Dissecting true epigenetic inheritance via gametes from effects of confounding factors in human studies is a major challenge and often not possible, mostly because progeny and parents usually share the same environment. Data are often evaluated and reported by the subjects themselves or their relatives and biological samples sometimes are not easily accessible. Furthermore, a comprehensive adjustment for potential confounding factors such as genetic and non-genetic family history, physical activity, dietary patterns and energy intake, culture, and behavior is usually not possible. More prospective studies on epigenetic inheritance of fasting and exercise in humans and combined studies in man and, for example, mouse are required to better understand the true potential of the beneficial impact on metabolic health in the progeny. Yet the existing epidemiological data agree with the more convincing data from animal models showing that acquired metabolic traits affect metabolic health in the filial and grand-filial generations.

### Animal models showing epigenetic inheritance across generations

Using rodents as model systems enables the exclusion of confounding factors and other limitations of human studies such as genetic and environmental heterogeneity. Within the last decade, many studies demonstrated that parental nutrients, trauma as well as other environmental factors can have profound effects on metabolic health across generations even when the external factor is no longer affecting the individual. The speedy increase of new discoveries also indicates the relevance of this thriving research field. However, the studies usually differ significantly in the type of diet, time and duration of exposure, phenotyping of the metabolic outcome, and in the analyses of underlying molecular mechanisms. In the following, we provide an overview of the current literature and structure it according to the type of dietary constraint, individual food supplements, and endocrine disruptors in rodents (see also Table 1).

However, because it is such a striking and convincing example of epigenetic inheritance, we want to start with a study that is not on the topic of an acquired metabolic trait but that demonstrated the epigenetic inheritance of a learned behavioral trait. In this case, the conditioned fear associated with the odor of acetophenone (in F0), was transmitted to filial (F1) and grand-filial generations (F2) (Dias and Ressler 2014). What makes this study so outstanding is the fact that the behavioral sensitivity to acetophenone in the F1 and F2 generations was accompanied by increased activity of the corresponding olfactory receptor (*Olfr151*) and the hypomethylation of the *Olfr151* gene in sperm of F0 and F1 males. Epigenetic inheritance via the parental gametes was evident due to the use of in vitro fertilization (IVF) and naïve and healthy fosters to produce F1 and F2 offspring (Dias and Ressler 2014). Besides the well-studied epigenetic mechanism of transgenerational inheritance of coat color associated with the agouti locus (Blewitt et al. 2006; Dolinoy et al. 2010) and a kinked tail phenotype with the axin locus in mice (Morgan et al. 1999), this is, so far, one of the most convincing associations between the epigenetic inheritance of a trait and a molecular mechanism—so convincing, because of its rational association with the expression and function of a single odor receptor gene.

Nevertheless, nutrition represents a major environmental factor that strongly impacts on metabolic health in humans and other mammals. Changes in the nutritional status can lead to acquired metabolic phenotypes that can be inherited across generations (Carone et al. 2010; Radford et al. 2014).

#### Undernutrition

Investigations on undernutrition mostly focus either on caloric restriction or protein-restricted (isocaloric) diets, both of which were shown to significantly increase longevity and impact behavioral traits in adult rodents (Ingram and Cabo 2017). Furthermore, caloric restriction could ameliorate cardiomyopathy through elevated levels of PPARα (Cohen et al. 2017) and completely prevent diabetes in New Zealand Obese (NZO) mice (Baumeier et al. 2015). These beneficial effects of caloric restriction on health parameters in adults must be distinguished from detrimental effects of exposure to dietary restriction in early life.

Maternal (F0) undernutrition by feeding isocaloric low protein diet (LPD) during gestation and lactation effectively altered in utero environment (Sayer et al. 2001) and significantly reduced milk production and milk quality (Crnic and Chase 1978; Grigor et al. 1987; Grimble and Mansaray 1987; Passos et al. 2000). In both male and female rats (F1), prenatal and early postnatal caloric restriction led to lower body weight with hypoglycemia at weaning (Desai et al. 2007). They were also at higher risk for developing obesity later in adulthood (Desai et al. 2007; Crossland et al. 2017). In addition, differential hepatic gene expression, decreased energy expenditure and increased anxiety-like behavior were found in these rats (Crossland et al. 2017; Balasa et al. 2011). Strikingly, maternal (F0) LPD either only during gestation or during gestation and lactation also had a profound effect in the F1 males on sperm quality, the structure or function of the epididymis, and was associated with increased testicular and sperm oxidative stress (Cavariani et al. 2019; Rodriguez-Gonzalez et al. 2014; Toledo et al. 2011). DNA methylation in offspring’s (F1) sperm was also altered after in utero (F0) caloric restriction (Radford et al. 2014). Although the proof of an intergenerational inheritance in the latter experiments is still missing, the observed changes in the male reproductive system and the sperm methylome following protein or caloric restriction in early life lend support to potential epigenetic mechanisms.

Whereas the studies above only linked early nutritional constraints to epigenetic modifiers, clear intergenerational epigenetic inheritance of metabolic impairment after in utero undernutrition was demonstrated as well (Jimenez-Chillaron et al. 2005, 2009; Kaczmarek et al. 2016). Restriction of maternal (F0) caloric intake to 50% of the caloric consumption in an ad libitum fed control group during lactation resulted in altered body composition and delayed onset of puberty in the offspring (F1). Transcriptomic changes affected kisspeptin signaling in the hypothalamus of F1 pups. These phenotypic and transcriptomic changes in the F1 were subsequently transmitted to both male and female F2 progeny in agreement with potential intergenerational inheritance of traits acquired before weaning (Kaczmarek et al. 2016). However, in this study only the hypothalamus was analyzed omitting other metabolically active tissues that could be important. Furthermore, low birth weight, obesity, and impaired glucose tolerance in male offspring (F1) after maternal (F0) caloric restriction (Jimenez-Chillaron et al. 2005) were transmitted to the subsequent and naïve (unchallenged) F2 mice through either paternal, maternal or both lineages (Jimenez-Chillaron et al. 2009). In contrast, the increase in the expression of hippocampal *Insulin-like growth factor 2* (*Igf2*) of adult rats after grand-maternal caloric restriction were transmitted only via the female germline (Harper et al. 2014).

Not only maternal but also paternal LPD was found to impact on the offspring’s metabolic pathways, indicating intergenerational inheritance more clearly. For instance, it was shown that paternal LPD before mating led to modest alterations in DNA methylation in offspring’s liver, and therefore, elevated expression of hepatic genes was involved in lipid and cholesterol biosynthesis (Carone et al. 2010). This intergenerational transmission of the information about paternal LPD was clearly attributed to epigenetic changes in spermatozoa due to the fact that IVF was used to produce the offspring (Sharma et al. 2016).

Evidence for transgenerational epigenetic inheritance of prenatal undernutrition is hard to find in the literature. In one example, prenatal protein restriction (F1) led to reduced insulin levels at weaning as well as reduced β-cell-to-pancreatic-mass ratio at birth and at weaning over three generations (F1–F3) via the maternal lineage. However, body weight at birth was only significantly lower in the first generation and by weaning all pups caught up in all generations (Frantz et al. 2011). Also altered expression of genes involved in DNA methylation and histone acetylation in three generations (F1-F3) after maternal (F0) caloric restriction was transmitted via the maternal lineage (Nowacka-Woszuk et al. 2018). Moreover, rats exposed to a diet restricted in protein and calories for over 50 generations displayed lower birth weight, a higher susceptibility to diabetes, and altered histone modification of the insulin promoter. This phenotype and associated epigenetic signatures were not reversed after two generations of unrestricted access to normal chow (Hardikar et al. 2015).

Taken together, several studies in rodents confirmed intergenerational epigenetic inheritance due to undernutrition. However, literature on the dissection of the underlying molecular mechanism(s) is still sparse (Radford et al. 2014; Sharma et al. 2016) and requires further research (see also “Epimutations triggered by diets” section).

#### Overnutrition

Maternal overnutrition, overweight, and diabetes before or during gestation notably increases the offspring’s risk for obesity and diabetes (reviewed in Alfaradhi and Ozanne 2011). Also the paternal influence on offspring’s metabolic health has become more and more apparent (reviewed in Rando 2016). In rodents, for example, the in utero exposure of embryos to maternal overnutrition has significant impact on progeny metabolism (Masuyama and Hiramatsu 2012; Buckley et al. 2005; Cannon et al. 2014). A series of studies showed that the offspring’s exhibition of hypertension, insulin resistance, and hyperlipidemia was associated with changes in DNA methylation and histone modification (see “Epimutations triggered by diets” section) (Masuyama and Hiramatsu 2012; Ge et al. 2014). Interestingly, this metabolic phenotype was further transmitted to granddaughters (F2) independent of the maternal diet (F1) but reversed after three consecutive generations of normal maternal diet suggesting an intergenerational mode of epigenetic inheritance (Masuyama et al. 2015). In another study, effects of maternal overnutrition were found on the gene expression levels but not on DNA methylation patterns in liver of male offspring (F1) (Cannon et al. 2014). Additional support for intergenerational epigenetic inheritance came from studies in which a maternal high-fat diet (HFD) before or during gestation (F0) resulted in significantly higher body weight and impaired glucose tolerance in offspring (F1) and during early life in the grand-filial generation (F2). In animals of the F1 and F2 generation, pancreatic islets were affected such that β-cell function and proliferation were impaired (Graus-Nunes et al. 2015; Huang et al. 2017).

In rats, a paternal HFD exposure (F0) impaired insulin secretion and glucose tolerance in female offspring (F1) (Ng et al. 2010). Gene expression signatures in pancreatic islets and in white adipose tissue were altered (). Also, cognitive impairments were found in the filial, but not grand-filial generation, of HFD-induced obese fathers accompanied by altered methylation patterns in paternal spermatozoa, suggesting inter- but not transgenerational inheritance (Zhou 2018). An obesogenic environment in both the paternal (F0) and filial (F1) generation exacerbated the obesity risk in male F1 animals thus indicating an additive effect of HFD treatment over two generations. The F1 generation exhibited increased body weight and adiposity as well as higher concentrations of serum cholesterol, triglyceride, HDL, and NEFA compared with the F1 and F0 generations on control diet (Fullston et al. 2015). Furthermore, paternal HFD-induced prediabetes increased the susceptibility of diabetes in offspring and altered gene expression in pancreatic islets as well as methylome patterns in paternal sperm (Wei et al. 2014). Unfortunately, in all these studies above in support of intergenerational inheritance of a paternal exposure, confounding factors from natural fecundation were not excluded by complementing the data with IVF-derived offspring.

Interestingly, observations regarding sex specificity and parental lineage of inheritance often differ between studies. Parental HFD led to increased body weight and glucose intolerance in a sex-specific and parent-of-origin-specific manner (Huypens et al. 2016). Moreover, intergenerational inheritance through the germline was evident because offspring were produced via IVF instead of natural fecundation. Here, gametes of HFD-treated parents were used and resulting embryos were transferred to healthy foster mothers to carry out the progeny. This use of IVF excluded that confounding factors during gestation and lactation, transfer of parental microbiome, or parental behavior could contribute to the observed epigenetic inheritance (Huypens et al. 2016). A significant increase in body length and reduced insulin sensitivity were reported in the F1 and F2 generation after maternal HFD exposure through both maternal and paternal lineages (Dunn and Bale 2009). However, in the F3 generation, this increased body size was only displayed by females and only via the paternal lineage (Dunn and Bale 2011). Similarly, it was confirmed recently that maternal HFD provoked obesity and insulin resistance, but additionally addictive-like behavior in offspring up to the third generation. Intriguingly, phenotypes in the third generation differed immensely between sexes, with males only displaying obesity and females only displaying addictive-like behaviors (Sarker et al. 2018).

Transgenerational transmission of grand-paternal overnutrition was revealed by a study showing that effects such as obesity and insulin resistance were transmitted to the F2 generation through both parental lineages (Fullston et al. 2013). Furthermore, HFD modulated the DNA methylation and transcriptional profile of testes and sperm microRNA content in F0 and F1 males (Fullston et al. 2013) and diminished reproductive health in the filial and grand-filial generations (Fullston et al. 2012). Preconceptional paternal HFD (F0) had a strong impact on offspring’s (F1) body weight and β-cell mass (de Castro et al. 2016) as well as on the hepatic transcriptomic and metabolic signatures of granddaughters (F2) with reduced hepatic cytokine and triglyceride levels (de Castro et al. 2019). Also overnutrition during early life of fathers (F0) had transgenerational effects on glucose tolerance in male F1 and F2 offspring (Pentinat et al. 2010).

Not only epigenetic marks in sperm but also alterations in the oocyte genome were found to play a role in the epigenetic inheritance across generations. A convincing example for transgenerational epigenetic inheritance was given by the transmission of a diet-induced cardiovascular phenotype to male F1, F2, and F3 offspring as well as female F1 and F2 offspring. Although the F0 maternal high-fat high-sugar diet exposure resulted in the transmission of damaged mitochondria via oocytes to the F1 animals, also F1 males transmitted the cardiovascular phenotype to their progeny (F2). The latter observation suggested that maternal mitochondria transmitted via oocytes did not represent the primary mode of inheritance. In addition, due to the use of IVF for the production of offspring, confounding factors were excluded (Ferey et al. 2019).

Taken together, it is now clearly established that parental HFD in rodents enhances the risk for obesity and glucose intolerance across generations (Ng et al. 2010; Wei et al. 2014; Huypens et al. 2016; Sarker et al. 2018; Fullston et al. 2013; de Castro et al. 2019). Compared to parental undernutrition, more studies showing transgenerational epigenetic inheritance after parental overnutrition can be found in the literature. However, only very few excluded confounding factors associated with natural fecundation (Ferey et al. 2019). It has also been demonstrated that paternal overnutrition is associated with epigenomic changes especially in DNA methylation and the expression and modification of small RNAs in spermatozoa (de Castro et al. 2016; Zhang et al. 2018; Chen 2016a). Although these studies in general demonstrate the adverse effects of parental overnutrition on metabolic health in the offspring, it was also shown in rodents that these consequences can be successfully ameliorated by parental diet and exercise interventions (McPherson 2015).

#### Nutritional supplements and endocrine disruptors

Increasing evidence suggests that not only macronutrients but also micronutrients play an important role in the epigenetic programming of the offspring’s health. Methyl donors such as folic acid and vitamin B12 in diets or nutritional supplements were shown to have strong effects on DNA methylation patterns and could impair metabolic and psychologic health (Shorter et al. 2015). Both folic acid deficiency as well as its high dose supplementation had detrimental effects on germ cell development, reproductive fitness, and altered DNA methylation in sperm of mice (Ly 2017). In rats, paternal methyl donor depletion for 5 weeks prior to mating also increased offspring’s depression- and anxiety-like behaviors (McCoy et al. 2018). A clear causative demonstration of effects from a dietary methyl donor supplementation was provided for the case of the mouse *Agouti viable yellow* (A^vy^) allele. The allele is hypermethylated in mothers that receive a diet supplemented with methyl donors. The resulting phenotype comprises a pseudoagouti coat color and protection against obesity and diabetes which is inherited epigenetically across generations (Cropley et al. 2006; Wolff et al. 1998).

Furthermore, synthetic chemicals in the environment that interfere with the hormonal system, so-called endocrine disruptors, can affect offspring’s health. As one of the first examples, it was shown that vinclozolin, a widely used fungicide with significant anti-androgenic effects, has transgenerational effects in rats. Maternal transient exposure to vinclozolin during pregnancy negatively affected male rats’ fertility from the F1 until the F4 generation through the male germline (Ben Maamar et al. 2018).

Another rather ubiquitous endocrine disruptor is bisphenol A (BPA). It is a precursor for the production of many different plastics used extensively, for example, to line water pipes and in diverse household products. Consequently, BPA is detectable in most humans (Vandenberg 2010). Long-term exposure to environmental doses of BPA might increase, for example, the development of chronic metabolic diseases and reproductive abnormalities. Studies in rodents suggested inheritance of adverse effects of BPA across generations (Manikkam 2013; Salian et al. 2009).

The list of endocrine disruptors and chemicals that directly impact on epigenetic mechanisms will most likely continue to grow. However, whether and to which extent they impact on epigenetic inheritance across generations still needs to be investigated. Also, it should be noted that chemicals such as endocrine disruptors are not exclusively limited to synthetic compounds but also occur naturally. For example, phytoestrogens are produced by a large number of plants including soy products, vegetables and fruits that humans always have consumed. Although phytoestrogens are absorbed into the human circulatory system, their physiological effects are rather unclear.

### Epimutations triggered by diets

Three main types of epimutations are currently considered as the major mediators of epigenetic inheritance: various post-translational histone modifications, genomic DNA methylation and diverse non-coding RNAs (Ben Maamar et al. 2018; Miska and Ferguson-Smith 2016). Commonly, these epigenetic markers can have profound effects on the transcriptional regulation with the ability to either enhance or repress gene expression.

#### Histone modifications

Post-translational histone modifications such as methylations and acetylations regulate the accessibility of DNA for transcription factors and can therefore regulate gene expression. Nevertheless, the role of histone modifications in transgenerational epigenetic inheritance is poorly understood and, so far, rarely investigated. One reason for this void lies in the complexity and dynamic nature of histone modifications. It is currently not possible to grasp a whole genome-wide picture of all histone modifications in one particular state. Secondly, in mammalian sperm, histones are widely replaced by protamines at a late stage in the haploid phase of spermatogenesis. Protamines allow for denser packaging of DNA, which is essential for sperm head condensation and DNA stabilization. This observation most likely excludes histones as major carriers of epigenetic marks in sperm (Ward and Coffey 1991). Yet, 10–15% of histones are retained in human sperm and 1–8% of histones remain in mouse sperm. Although, the fact that diets and nutrition affect histone modifications is established (reviewed in Molina-Serrano et al. 2019), the extent to which they may contribute to epigenetic inheritance across generations still requires further investigation (Hammoud 2009). Some recent publications suggested that the in utero exposure to maternal HFD treatment led to alterations in histone acetylation and methylation in the filial (Masuyama and Hiramatsu 2012) and grand-filial generation’s (Masuyama et al. 2015) adipose tissue and differential expression of the adiponectin and leptin genes.

#### DNA methylation

As already mentioned above DNA methylation patterns in mammals are globally erased twice. This so-called reprogramming occurs first during the initial cell divisions after fertilization of the oocyte and has been linked to the totipotency of embryonic cells (Reik et al. 2001). The second round of global DNA demethylation occurs in primordial germ cells later during embryogenesis. However, it has also been shown that imprinted genes and genes that are differentially methylated between maternal and paternal alleles can be protected against reprogramming and can thus escape global demethylation (Lane 2003; Morgan et al. 2005). Therefore, DNA methylation is a potential candidate for epigenetic marks that might impose epigenetic inheritance across generations. Accordingly, patterns of DNA methylation have been analyzed, for example, in sperm and other tissues in transgenerational studies. Although several associations between transgenerational epigenetic inheritance and methylation changes were found, they do not prove that these epimutations are causative (Dias and Ressler 2014; Carone et al. 2010). For example, in humans, paternal obesity altered DNA methylation in offspring (Soubry et al. 2015) and in mice, early caloric restriction and paternal LPD could alter DNA methylation in offspring’s sperm and tissues (Carone et al. 2010; Radford et al. 2014). In addition, DNA methylation patterns of metabolic genes were altered in mothers and offspring following maternal HFD treatment (Ge 2014). For example, methylation of the *Leptin* promoter was increased and methylation of the *Ppara* promoter was decreased in oocytes of HFD-treated mice (F0) as well as in oocytes and liver of their female offspring (F1) (Ge 2014). What is lacking, so far, is a causative proof, for example, showing that the experimental manipulation of distinct methylation patterns associated with nutrition or diets would either cause or prevent epigenetic inheritance across generations. There are also a number of studies claiming that, for example, changes in sperm methylome were not accountable for the epigenetic inheritance of diet-induced phenotypes (Shea 2015) or other papers that did not observe global DNA methylome changes in the progeny’s tissues following in utero caloric restriction (Zelko et al. 2019; Zhu et al. 2016).

#### Noncoding RNAs

Small non-coding RNAs (sncRNAs) such as microRNAs (miRNAs), small-interfering RNAs (siRNAs) and piwi-interacting RNAs (piRNAs), have been recognized as functional regulators of gene transcription and translation, for example, by targeting messenger RNAs (mRNAs) for degradation. Apparently, also other non-coding RNAs such as ribosomal RNAs (rRNAs) and transfer RNAs (tRNAs) as well as their fragments function as regulators of gene expression. Therefore, non-coding RNAs were suggested as a third group of epigenetic marks that might transmit an environmentally induced phenotype from parents to subsequent generations (Chen et al. 2016b).

In contrast to DNA methylation and histone modifications, there is rather convincing data suggesting a causative role for sncRNAs in the epigenetic inheritance of nutrition related phenotypes. So far, most of the studies investigated the male contribution to epigenetic inheritance and therefore only sncRNAs of sperm and not oocytes (Zhang 2019). Additionally, considering the enormous difference in size and RNA content between oocyte and sperm cell, the question was raised how effectively a fraction of sperm RNA can impact the F1 epigenome and phenotype (Kiani and Rassoulzadegan 2013). Clearly, alterations in sperm sncRNA content after paternal HFD and obesity were documented in several studies (Donkin et al. 2016; Fullston et al. 2013; Sarker et al. 2019). Sperm-derived small RNAs and in particular fragmented tRNAs were altered by paternal LPD in mice and were sufficient to regulate expression of endogenous retroelements in the preimplantation embryo. This was the case following injection of small RNA fractions isolated from mature sperm or of synthetic fragmented tRNAs into oocytes (Sharma et al. 2016). There is also evidence that vesicles, so-called epididymosomes which carry RNAs from somatic cells, fuse with sperm cells during their maturation and migration through the epididymis (Sharma et al. 2016). Also long coding and non-coding RNAs were altered in sperm of HFD-induced obese fathers and their offspring (An 2017). Several additional intriguing gain-of-function experiments have further consolidated that either specific miRNAs or fractions of RNAs isolated from sperm are sufficient to reproduce the outcome of epigenetic inheritance of a paternal nutritional restriction or challenge. Synthetic oligos or fractions of sncRNAs were injected into naïve zygotes and reproduced respective metabolic and behavioral phenotypes in the offspring (Chen 2016a; Sarker et al. 2019; Gapp 2014; Grandjean 2015). In order to demonstrate that the examined small RNAs are also required in vivo for epigenetic inheritance, it would be essential to experimentally eliminate (or repress) these RNAs in gametes and assess whether this leads to the loss of the phenotype in the resulting offspring. Towards this end it has already been shown that deletion of the tRNA methyltransferase, *Dnmt2*, in mice abolished the epigenetic inheritance of HFD-induced metabolic disorders to offspring. This was accompanied by a loss of m^5^C-methylation in position 38 of some tRNAs and an increase of fragmented tRNA-Gly in sperm (Zhang et al. 2018).

Thus, there is persuasive evidence that non-coding RNAs play an important role in epigenetic inheritance of acquired metabolic phenotypes via sperm in rodents. Similar data for oocytes are still missing and the relevance for humans is awaiting formal proof. Also it should probably be pointed out that at least formally the contribution of acquired *genetic* alterations, so far, cannot be ruled out, since none of the experimental approaches, included genome-wide sequencing across generations yet. However, the (fast) reversion of epigenetically inherited phenotypes may possibly be used as argument against a genetic contribution.

### Therapeutic interventions

Do we have the option to avoid the transmission of potentially harmful epigenetic changes to our offspring? So far, there are only few studies dealing with this topic. Pharmacological compounds targeting epigenetic mechanisms are already in clinical use for cancer therapy. In diabetes, metformin is a widely used antidiabetic drug with potent glucose-lowering activity and it was recently documented that it also modulates epigenetic processes (Bridgeman et al. 2018). Studies of the impact of maternal metformin on the offspring are limited to mothers with gestational diabetes and mostly report highly variable results regarding offspring’s body weight and metabolic health outcome (Carlsen et al. 2012; Ijas et al. 2015; Salomaki et al. 2013). Bariatric surgery is a highly effective intervention for weight loss and metabolic improvement in obese patients. Several studies in humans and rodents after maternal gastric bypass, however, yielded inconsistent results regarding the offspring’s metabolic health (Carreau et al. 2017; Pietrobon et al. 2019; Willmer et al. 2015). Furthermore, parental physical exercise can have a significant impact on the offspring. Maternal exercise during pregnancy was linked to reduced obesity risk in offspring and differentially methylated regions in imprinted genes in the offspring (McCullough et al. 2015). However, these observations could also be attributed to the in utero exposure of the embryo. Recently, different circulating miRNAs in blood that are controlled by exercise and genome-wide DNA methylation changes were detected in human spermatozoa providing a relevant indication of intergenerational epigenetic inheritance (Stanford et al. 2018)—somewhat reminiscent to the mechanism described for epididymosomes in rodents (see above). Once the mechanism(s) of epigenetic inheritance become more established the issue of blocking the transmission of undesired traits can be addressed more easily.

## Conclusion

From reviewing the current literature there is plenty of evidence from animal studies and strong support from human prospective and epidemiological studies in favor of intergenerational epigenetic inheritance of metabolic disorders following dietary constraint. Results for transgenerational epigenetic inheritance, however, vary strongly depending on the experimental setup and are sometimes ambiguous. Human studies are always influenced by cultural and direct environmental factors and therefore more difficult to interpret. Confounding factors such as behavior learned from parents and the social context, shared microbiomes, heterogeneous genetic and environmental backgrounds and others are difficult to discern from epigenetic inheritance transmitted via gametes in humans.

Certainly, direct in utero exposure and early postnatal exposure through lactation to maternal diet also can have profound effects on metabolism later in life. In addition, paternal and maternal nutrition before fertilization also affects metabolic health of the offspring. In experimental designs it is therefore important to exclude confounding factors, for example, by using IVF and healthy foster mothers. This way, true epigenetic inheritance through the parental gametes and potential mechanisms can be investigated. Interestingly, although both parents influence their offspring, the mode of action and the specific metabolic consequences for the offspring apparently vary between maternal and paternal epigenetic inheritance. In man overnutrition, obesity and diabetes are strong factors for the offspring’s metabolic health and make parents to some degree responsible for the health of their offspring. In this sense we have learned that not all inheritance is fatefully determined by the genes we inherited from mom and dad. Instead the discovery of epigenetic inheritance across generations brings some freedom into inheritance via gametes that is controlled by parental lifestyle. Unfortunately, we are still far from predicting the best type of intervention—be it exercise, change of diet or pharmacological intervention—for each individual parent to provide the best conditions for metabolic health of our offspring. Yet, we are positive that current and future research of epigenetic inheritance will generate the basic knowledge to make good decisions.
